# Assessing the impact of choosing different deformable registration algorithms on cone-beam CT enhancement by histogram matching

**DOI:** 10.1186/s13014-018-1162-3

**Published:** 2018-11-07

**Authors:** Halima Saadia Kidar, Hacene Azizi

**Affiliations:** Department of Physics, Ferhat Abbas Setif University, El Bez Compus, 19000 Setif, Algeria

**Keywords:** CBCT images, Deformable registration, Histogram matching, Adaptive radiation therapy

## Abstract

**Background:**

The aim of this work is to assess the impact of using different deformable registration (DR) algorithms on the quality of cone-beam CT (CBCT) correction with histogram matching (HM).

**Methods and materials:**

Data sets containing planning CT (pCT) and CBCT images for ten patients with prostate cancer were used. Each pCT image was registered to its corresponding CBCT image using one rigid registration algorithm with mutual information similarity metric (RR-MI) and three DR algorithms with normalized correlation coefficient, mutual information and normalized mutual information (DR-NCC, DR-MI and DR-NMI, respectively). Then, the HM was performed between deformed pCT and CBCT in order to correct the distribution of the Hounsfield Units (HU) in CBCT images.

**Results:**

The visual assessment showed that the absolute difference between corrected CBCT and deformed pCT was reduced after correction with HM except for soft tissue-air and soft-tissue-bone interfaces due to the improper registration. Furthermore, volumes comparison in terms of average HU error showed that using DR-NCC algorithm with HM yielded the lowest error values of about 55.95 ± 10.43 HU compared to DR-MI and DR-NMI for which the errors were 58.60 ± 10.35 and 56.58 ± 10.51 HU, respectively. Tissue class’s comparison by the mean absolute error (MAE) plots confirmed the performance of DR-NCC algorithm to produce corrected CBCT images with lowest values of MAE even in regions where the misalignment is more pronounced. It was also found that the used method had successfully improved the spatial uniformity in the CBCT images by reducing the root mean squared difference (RMSD) between the pCT and CBCT in fat and muscle from 57 and 25 HU to 8HU, respectively.

**Conclusion:**

The choice of an accurate DR algorithm before performing the HM leads to an accurate correction of CBCT images. The results suggest that applying DR process based on NCC similarity metric reduces significantly the uncertainties in CBCT images and generates images in good agreement with pCT.

## Background

In the past decade, on board cone-beam CT, integrated into linear accelerators was frequently used for image guidance of radiotherapy. It allowed the verification and the correction of patient’s setup during the course of treatment in three dimensions with sufficient soft tissue contrast and low patient dose [1–4]. Therefore, it became a powerful tool for improving tumor targeting and reducing dose delivery to normal tissues [5].

**Fig. 1:**
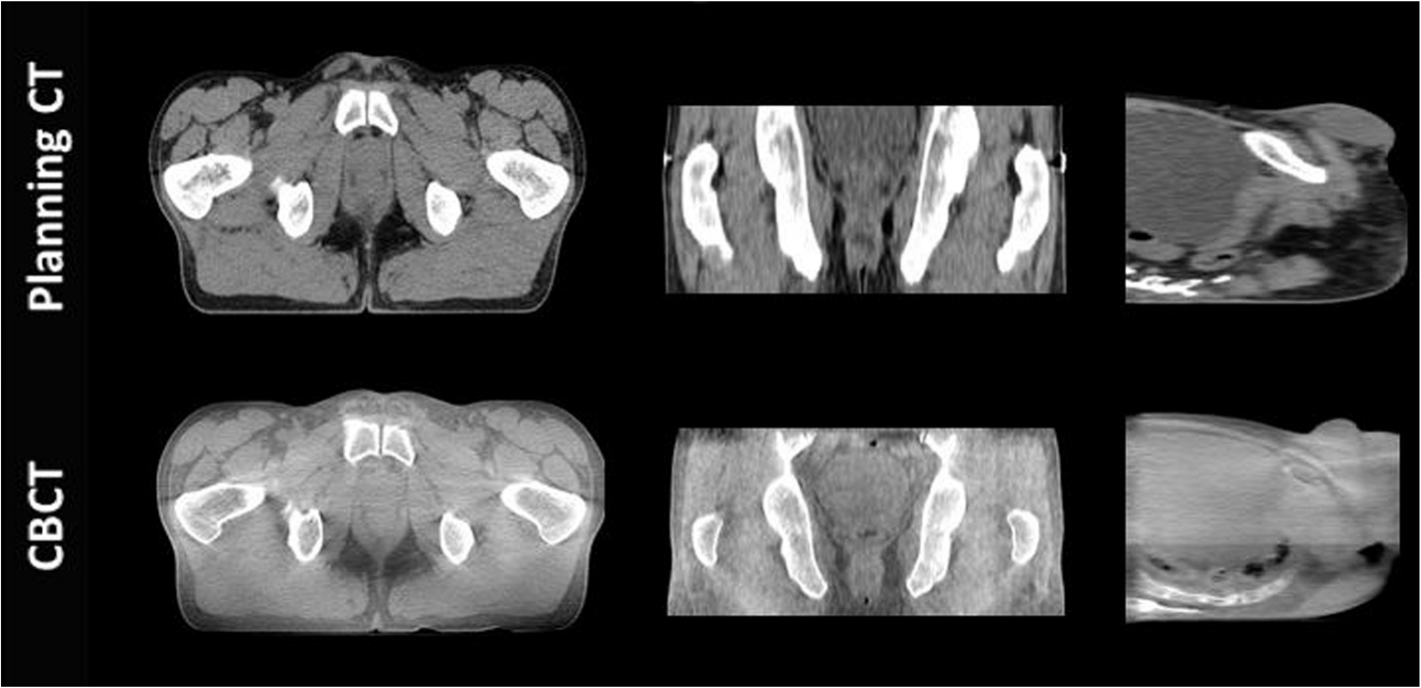
pCT and CBCT for the same patient (axial, coronal and sagital views) displayed using the same window level

Recently, the development of CBCT systems in terms of images acquisition, rapidity and improved image quality has underlined the question of using CBCT images for adaptive radiation therapy (ART). This technique aims to adapt the treatment planning with patient anatomy modification throughout the entire treatment; it is mainly based on three complex and consuming time processes: acquisition of daily CBCT images for making decision if the re-planning is necessary by comparing them to the CT images, the second process concerns the acquisition of new pCT images and the delineation of volumes of interest to provide a base for the last process which is the dose re-calculation [6]. However, repeated acquisition of CT images for each planning is unjustifiable, due to the accumulated dose. In addition, the preposition of using daily CBCT images directly for dose calculation is limited, owing to their reduced contrast compared to CT images, as shown in Fig. 1, and the large variation of Hounsfield Units caused by the increased amount of scattered radiation [7, 8].

Despite these drawbacks, several studies investigated the feasibility of CBCT images for dose calculation proposing three main “pCT-based” approaches to correct the HUs distribution and minimize as possible the density differences between CBCT and pCT to ensure an accurate dose calculation based on CBCT images. The first approach, known as HU mapping, consists of replacing the HUs values in CBCT by their equivalent points in pCT after the application of rigid or deformable registration. The accuracy of this approach is strongly dependent on region of body in which it is applied and it is available just for regions where the intra-scan motion and organs deformation are insignificant [9, 10]. The second approach is the Multilevel Threshold (MLT), it classifies all CBCT pixels with similar HUs into three or four different segments based on pCT. The use of such approach showed a high accuracy especially when combined with DR which minimizes the effect of organs deformation [11–14]. The last approach is the histogram matching (HM) which allows the adjustment of HUs values between CT and CBCT using cumulative histograms. This modification yielded a good agreement between CT and modified CBCT even for breast and prostate cancer where the intra-scan motion and organs deformation are significant [13, 15]. Other correction categories can be found in literature such as: “Scatter calibration” and “physics-based” techniques, which aim to directly use CBCT images without recourse to pCT-based strategies using empirical look-up-table (LUT) to calibrate CBCT images [16–18] and scatter measurement or simulation [19–23].

Focusing on pCT-based techniques, the previously cited works showed that the correction accuracy depends on the correspondence between the voxels of CBCT and pCT images. Therefore, the choice of DR algorithm must be validated.

The present paper aims to evaluate the impact of using different DR algorithms on the accuracy of CBCT enhancement by HM. A dataset containing CT and CBCT images for patients with prostate cancer was used to generate corrected CBCT, then, HUs values were compared for corrected CBCT and original CT using different metrics.

The remainder of this paper is organized as follows. In section “Methods and Materials”, used data and each step to correct CBCT images are described. Numerical results based on ten prostate cancer patient data sets are presented in section “Results”. In section “Discussion”, we further discuss the performance and the effect of DR on the correction quality, and finally conclude the paper in section “Conclusion”.

## Methods and materials

### Data description

This study was performed on data sets of 10 patients with prostate cancer containing pCT and CBCT images obtained by GE CT scan (General Electric Medical Systems) and on-board imager (OBI, Varian Medical Systems) mounted on the gantry of clinical iX21 linear accelerator, respectively. The settings of pCT and CBCT acquisition according to pre-defined protocols are recapitulated in Table 1. The slices number differed from a patient to another; it ranged from 123 to 159 slices in the pCT images and from 50 to 64 slices in the CBCT images giving sufficient information about the anatomical distribution and the motion artifact variations. For all these data CBCT images were acquired for the first day of treatment to minimize the error of patient’s setup under the treatment machine. Since this technique is newly integrated in the clinical practice, the number of patients used in this study is limited.

**Table 1:** Acquisition settings

Protocol	Tube current (mA)	Exposure time (ms)	Tube voltage (kVp)	Axial image size (pixels)	Voxel size (mm^3^)
CT	360	500	100	512 × 512	0.8496 × 0.8496 × 3
CBCT	80	8632	125	512 × 512	0.8789 × 0.8789 × 2.5

### Images pre-processing

Initially, collected CBCT and pCT contained not only the information describing the patient’s body but also the couches of the CT scanner and the linear accelerator. For that reason, all images were pre-processed using the FIJI software [24] to select the region including the patient volume and remove the couches. Furthermore, to eliminate all unnecessary content, a fixed threshold was applied to assign all pixels outside the body surface (below − 700 HU for pCT and bellow -600HU for CBCT) to standard CT value for air (-1000HU) using the 3D Slicer software [25].

### Corrected CBCT generation

**Fig. 2:**
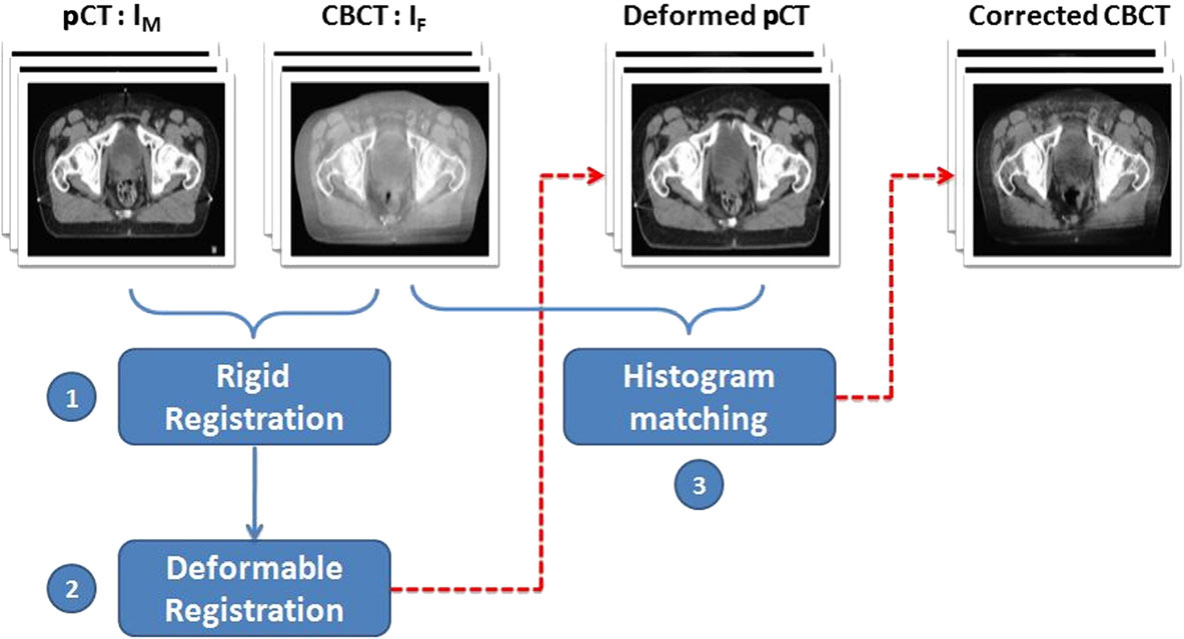
Workflow of corrected CBCT generation

In order to assess the impact of DR on the quality of CBCT enhancement, three intensity-based algorithms with different similarity metrics implemented in Elastix [26] were used. The workflow of corrected CBCT generation is described in Fig. 2.

Before starting the DR, the data sets for each patient were aligned using rigid 3D transformation with mutual information similarity metric (step1 in Fig. 2). Moreover, to minimize the effect of difference in organ deformation between pCT and CBCT, a multi-resolution B-Spline transformation including 3 levels was performed (step2 in Fig. 2). It is mainly based on the displacement of control points around a control point grid that is put on fixed image, according to the considered similarity metric [26]. The B-Spline interpolator was used to estimate iteratively the deformation field in these points and at each iteration the control points displacement was optimized using the adaptive stochastic gradient descent (ASGD). In this DR process, three similarity metrics were considered: the Normalized Correlation Coefficient (NCC), the Mutual Information (MI), and the Normalized Mutual Information (NMI).

The Normalized Correlation Coefficient (NCC) is given by:

1 $$ NCC\left({I}_F,{I}_M\right)=\frac{\sum_{x_i\in {\varOmega}_F}\left({I}_F\left({x}_i\right)-\overline{I_F}\kern0.15em \right)\left({I}_M\left(T\left({x}_i\right)\right)-\overline{I_M}\kern0.15em \right)}{\sqrt{\sum_{x_i\in {\varOmega}_F}{\left({I}_F\left({x}_i\right)-\overline{I_F}\kern0.15em \right)}^2\Big({I}_M\left(T\left({x}_i\right)\right)-\overline{I_M}\kern0.10em \Big){}^2}} $$

With *I*_*F*_ the fixed image, *I*_*M*_ the moving image using a given transformation *T* and |*Ω*_*F*_| is the number of voxels of the fixed image. $\overline{I_F}$ and $\overline{I_M}$ are the average gray values for the fixed and the moving images respectively.

The Mutual Information (MI) is defined as:

2 $$ MI\left({I}_F,{I}_M\right)=H\left({I}_F\right)+H\left({I}_M\right)-H\left({I}_F,{I}_M\right) $$

Where:$$ H\left({I}_F\right)=-\int {p}_{I_F}(a)\mathit{\log}{p}_{I_F}(a) da $$ and $$ H\left({I}_M\right)=-\int {p}_{I_M}(b)\mathit{\log}{p}_{I_M}(b) db. $$

With: H(I_F_) and H(I_M_) the entropies of I_F_ and *I*_*M*_ respectively. ${p}_{I_F}(a)$ and ${p}_{I_M}(b)$ are the pixel’s probabilities with values *a* and *b* in *I*_*F*_ and *I*_*M*_ respectively. *H*(*I*_*F*_, *I*_*M*_) is the joint entropy of *I*_*F*_ and *I*_*M*_.

The NMI is given by:

3 $$ NMI\left({I}_F,{I}_M\right)=1+\frac{MI\left({I}_F,{I}_M\right)}{H\left({I}_F,{I}_M\right)}=\frac{H\left({I}_F\right)+H\left({I}_M\right)\ }{H\left({I}_F,{I}_M\right)} $$

After DR, the 3D slicer software [25] was used to match the histograms of the CBCT images against the corresponding deformed pCT (step3 in Fig. 2). This processing method aims to adjust the HU values between pCT and CBCT images using their cumulative histograms. Each pixel value in the CBCT images is replaced by the HU having the same cumulative value in the pCT images according to the following formula:

4 $$ CBCT\left({H}_1\right)= pCT\left({H}_2\right) $$

Where *CBCT*(*H*_1_) represents the HU values for CBCT and *pCT*(*H*_2_) represents the HU values for pCT [13, 15].

### Data analysis

To evaluate the quality of corrected CBCT, deformed pCT images were considered as a reference for each patient. A visual assessment was performed by the calculation of absolute difference between pCT and CBCT images before and after HM to assess the discrepancies between them.

Furthermore, to evaluate quantitatively the agreement between corrected CBCT and pCT, three methods were used. The first one consists of the average HU error estimation over the entire volume [27] given by:

5 $$ {V}_{err}=\sqrt{mean\ \left({\left[{HU}_{pCT}\left(x,y,z\right)-{HU}_{CBCT}\left(x,y,z\right)\right]}^2\right)} $$

The second method is the Mean Absolute Error (MAE) plots creation which allows comparing the different tissue classes. It is based on the calculation of the MAE between pCT and corrected CBCT in equidistant bins across the HU scale. For this comparison a size of 20 HU was taken for each bin and the formula describing the MAE is given by:

6 $$ MAE=\frac{1}{N}\sum \limits_0^N\mid {HU}_{pCT}-{HU}_{CBCT}\mid $$

Where N is the number of pixels having intensities in [HU-10, HU + 10] in the pCT [28].

The third one is the image quality evaluation in terms of spatial uniformity. For this method, the mean pixel value among five regions of interest (ROIs) having 10 by 10 pixels and positioned in regions of the same soft tissue area is measured [29]. Then, the RMSD between the mean pixel values in the pCT and the CBCT images before and after correction are calculated.

## Results

### Visual assessment

**Fig. 3:**
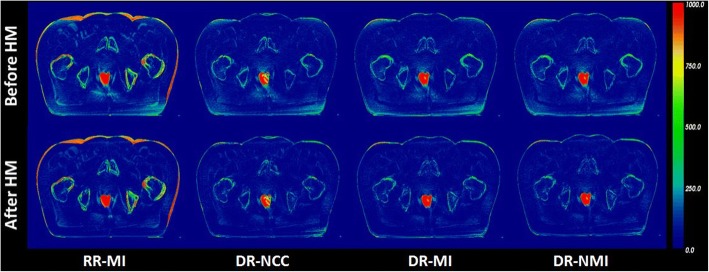
Absolute difference between deformed pCT and CBCT in the first row and corrected CBCT in the second row using one RR and three DR algorithms. Blue colors represent low discrepancies while red colors represent the highest ones

Figure 3 shows the absolute difference between deformed pCT and CBCT images for one patient before and after HM using three DR algorithms (DR-NCC, DR-MI and DR-NMI). Obtained results for a RR algorithm are also included to confirm the effect of morphologic deformation between pCT and CBCT on the quality of correction. The effect of applying HM is clearly visualized; it reduced the amount of artefacts in CBCT and yielded corrected images in good agreement with deformed pCT. However, high differences in bony regions and soft tissue-air interfaces are present due to the misalignment between CBCT and pCT.

### Volumes comparison

Results of volumes comparison for each patient in terms of HU average error between deformed pCT and CBCT before and after correction are shown in Table 2. The mean and the standard deviation are also presented.

**Table 2:** HU average error values between deformed pCT and CBCT before and after HM for each patient with the mean and the standard deviation

V_err_ [HU]								
Patient’s number	RR-MI		DR-NCC		DR-MI		DR-NMI	
	Before HM	After HM	Before HM	After HM	Before HM	After HM	Before HM	After HM
1	138.50	141.33	71.03	64.70	75.94	68.07	73.53	65.67
2	140.00	143.01	57.89	49.10	60.77	51.76	59.90	49.87
3	260.62	288.10	73.58	65.17	76.33	66.71	75.09	64.84
4	257.67	274.16	65.76	55.10	71.23	57.05	68.51	54.03
5	239.04	267.54	67.48	55.73	73.34	59.89	71.44	58.02
6	258.85	269.82	82.09	77.88	87.37	80.24	86.24	79.04
7	171.96	195.80	57.74	47.26	60.12	48.18	58.90	46.26
8	193.20	194.09	54.89	45.74	58.68	47.75	57.23	46.03
9	153.75	173.61	51.82	45.90	56.13	51.47	54.33	49.09
10	251.15	269.41	59.29	52.92	62.16	54.96	60.62	53.01
**Mean**	206.47	**221.68**	64.15	**55.95**	68.20	**58.60**	66.57	**56.58**
**SD**	52.21	**57.97**	9.50	**10.43**	10.12	**10.35**	10.06	**10.51**

The largest magnitude of V_err_ is observed for unprocessed CBCT images especially when using RR process where the mean HU error value was about 206.47 ± 52.21 HU. For the DR process, a significant decrease was obtained with error values ranging from 64.15 ± 9.50 to 68.20 ± 10.12 HU which confirms the performance of DR algorithms. Whereas, after the correction of CBCT images reduced values of HU of about 55.95 ± 10.43 HU, 56.58 ± 10.51 HU and 58.60 ± 10.35 HU were obtained for DR-NCC, DR-NMI and DR-MI, respectively, indicating that the HM after using DR-NCC yielded corrected CBCT images in good agreement with pCT images compared to unprocessed CBCT images.

### Tissue class’s comparison

**Fig. 4:**
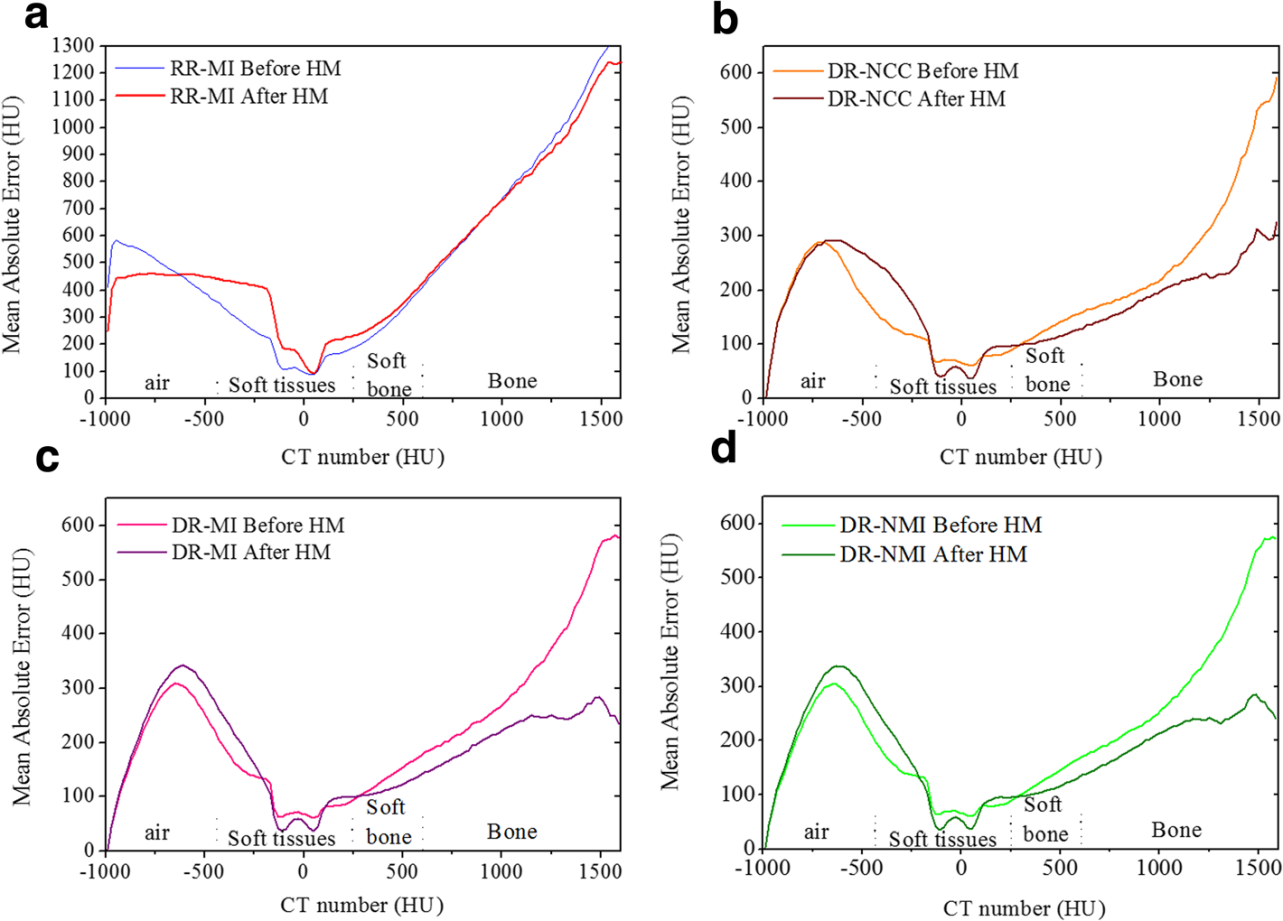
MAE values of CBCT images before and after correction using **a**) RR-MI, **b**) DR-NCC, **c**) DR-MI and **d**) DR-NMI

Since volumes comparison may not give information about the presence of large errors and their location, the MAE plots for each algorithm over the HU scale are illustrated in Fig. 4. Similarly to [13], HU scale for pCT images was divided according to the tissue type on different classes. All the values lower than − 400 HU was considered as air. The HU values between − 400 and 250 HU were associated to soft tissues, while those between 250 and 600 HU presented the soft bone. The remaining values (higher than 600 HU) were considered as bone.

Figure 4a compares MAE results using RR algorithm before HM with those obtained after HM. It shows obviously that the use of HM with RR increases the uncertainties in CBCT images, due to the misalignment between pCT and CBCT images. However, in (Fig. 4b, c, d) the MAE becomes lower and the combination of DR with HM contributes significantly to reduce the errors after correction, especially for pixels with CT number higher than 200 HU. For the values below − 200 HU a mismatch is observed and the MAE values after correction are higher than before. This is due to the low number of pixels in corrected CBCT containing the same HU values as pCT in the interfaces soft tissue-air, which is in agreement with the visual assessment where high errors were noticeable in those regions owing to the improper registration.

**Fig. 5:**
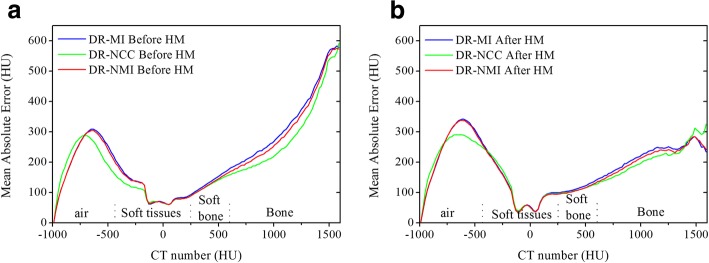
MAE values of CBCT images for each algorithm before HM (**a**) and after HM (**b**)

The DR performance comparison is depicted in Fig. 5. Plotting together the MAE values before and after correction against each other (Fig. 5a and b respectively) shows that the use of DR based on NCC metric was better than MI and NMI especially in soft tissue-air interfaces. Moreover, using the NCC metric in combination with HM produced more accurate CBCT images.

Concerning the uniformity of resulted CBCT images, the RMSD of the mean pixel values of ROIs between CBCT, corrected CBCT and pCT images are summarized in Table 3. The obtained results showed that the use of HM reduced the RMSD in fat and muscle (soft tissues) from about 57 and 25 HU to 8 HU, respectively, indicating that the CBCT image quality was brought closer to the pCT image quality through this correction technique.

**Table 3:** Comparison of the mean pixel values in fat and muscle between the pCT, CBCT and corrected CBCT images with the three different DR algorithms

Mean pixel values [HU]					
Patient’s number	Fat				
	pCT	CBCT	CBCT_DR-NCC_	CBCT_DR-MI_	CBCT_DR-NMI_
1	− 108.16	− 144.37	−90.73	−92.50	−92.06
2	−87.68	− 163.92	−84.98	−88.25	−85.77
3	−104.83	− 139.75	−90.03	−91.48	−91.16
4	− 103.80	− 151.60	−104.64	− 105.23	−105.19
5	−103.43	− 170.34	− 102.28	−103.16	−102.89
6	−109.43	− 130.55	−98.50	− 99.30	−99.09
7	−97.70	− 148.56	−97.08	−98.47	−97.97
8	−108.62	− 213.92	− 108.76	− 108.53	−108.04
9	− 110.23	−160.35	− 105.87	− 106.76	−106.82
10	−99.50	−123.33	−100.24	− 101.55	−101.40
**RMSD**		**56.83**	**8.19**	**7.38**	**7.57**
	Muscle				
1	49.45	−1.37	31.76	30.62	30.68
2	51.61	25.39	42.09	41.12	41.68
3	47.59	59.00	53.25	54.53	53.25
4	42.17	45.81	50.65	51.61	50.93
5	48.87	11.70	47.66	47.66	47.65
6	49.68	40.03	50.09	50.78	50.25
7	49.73	28.39	45.44	46.12	46.01
8	46.46	13.39	36.65	39.04	39.34
9	48.85	56.30	52.18	51.95	51.86
10	49.44	51.98	45.97	47.25	46.40
**RMSD**		**25.49**	**7.72**	**8.28**	**8.02**

## Discussion

In this work, the impact of choosing different registration algorithms on the quality of CBCT correction by HM was studied. One RR algorithm based on MI similarity metric and three DR algorithms including NCC, MI and NMI similarity metrics were validated.

Several studies investigated the accuracy of dose calculation based on corrected CBCT using HM with DR based on MI [13, 15] but our strategy differs from those studies because it aims to initially choose the appropriate DR algorithm, and then generate corrected CBCT images.

All the results confirmed that the performance of DR of each algorithm is strongly dependent on the region in which the transformation was applied. It was shown that all DR algorithms provided a good alignment between anatomical structures in pCT and CBCT compared to RR registration but their reduced ability to align some regions as soft tissue-air and soft tissue-bone interfaces was clearly visualised. In addition, the sensitivity of HM process to the quality of registration has been proved. It has been found that the better the alignment the more significant is the HM contribution to correct the HU distribution in CBCT images. For that reason, the best compromise for this correction method seems to be the use of DR with NCC similarity metric for which the MAE values after correction were found to be the lowest as indicated in Fig. 5. Also, this choice can be justified by Table 2 where reduced HU errors in corrected CBCT were obtained for the DR-NCC algorithm.

Despite the influence of DR accuracy on the HM process, the use of DR-NCC before HM yielded acceptable HU errors values compared to other studies investigating pCT-based approaches and direct approaches without recourse to pCT [29, 30]. In [29], Kida et al. applied a deep convolutional neural network (DCNN) method to improve the quality of CBCT images acquired for 20 prostate cancer patients. They reported that the RMSD of the mean pixel values for corrected CBCT images was about 11 and 14 HU in fat and muscle, respectively, while in our study the same evaluation showed that the RMSD was about 8 HU. This suggests that our proposed workflow had successfully improved the spatial uniformity in the CBCT images. Besides, Poludniowski et al. [30] studied for 12 patients (6 brain, 3 prostate and 3 bladder cancer patients) four correction methods based on “scatter calibration” and “scatter measurement” using CBCT images acquired by other linear accelerator (Elekta Linac). They reported that for prostate cancer the average HU error for each method, called also Root Mean Squared Difference, were about 95.5, 91.5, 73.1 and 67.7 HU. Whereas, in our study the HM considered as pCT-based approach resulted in average HU error of about 55.95 ± 10.43 HU when using DR-NCC indicating that although our CBCT images differs from theirs; our results are better than their findings.

To improve the correctness of the proposed workflow, the minimization of its limitations as the existence of non-comparable regions in the pCT and CBCT images, e.g. regions of gas in the rectum, is a priority. Thus, further investigations taking into account the correction of these regions before performing DR and HM are required. In addition, dosimetric evaluation is needed to validate the efficiency of using corrected CBCT for dose calculation in the context of adaptive radiation therapy. Also, we are looking forward to applying this workflow on large number of patients and translate it to other body regions.

## Conclusion

In this study, the impact of using different DR algorithms on the HM process to correct CBCT images was evaluated. The results showed that the quality of correction is strongly dependent to the accuracy of DR process and revealed that performing HM after DR with the NCC similarity metric contributed significantly to reduce the uncertainties in CBCT images. On the basis of this study, a combination of the present workflow with automatic segmentation algorithms could be a promising way towards online adaptive radiation therapy.

## Abbreviations

ART: Adaptive Radiation Therapy
ASGD: Adaptive Stochastic Gradient Descent
CBCT: Cone Beam Computed Tomography
DCNN: Deep Convolutional Neural Network
DR: Deformable Registration
DR-MI: Deformable Registration based on Mutual Information similarity metric
DR-NCC: Deformable Registration based on Normalized Correlation Coefficient similarity metric
DR-NMI: Deformable Registration based on Normalized Mutual Information similarity metric
HM: Histogram Matching
HU: Hounsfield Unit
LUT: Look Up Table
MAE: Mean Absolute Error
MLT: Multilevel Threshold
OBI: On-Board Imager
pCT: Planning Computed Tomography
RMSD: Root Mean Squared Difference
ROIs: Regions of Interest
RR-MI: Rigid Registration based on Mutual Information similarity metric
V_err_: Average HU error over the entire volume
